# Neurosymbolic AI as an antithesis to scaling laws

**DOI:** 10.1093/pnasnexus/pgaf117

**Published:** 2025-05-20

**Authors:** Alvaro Velasquez, Neel Bhatt, Ufuk Topcu, Zhangyang Wang, Katia Sycara, Simon Stepputtis, Sandeep Neema, Gautam Vallabha

**Affiliations:** Department of Computer Science, University of Colorado Boulder, 430 UCB, 1111 Engineering Dr, Boulder, CO 80309, USA; Oden Institute for Computational Engineering and Sciences, University of Texas at Austin, 201 E 24th St, Austin, TX 78712, USA; Oden Institute for Computational Engineering and Sciences, University of Texas at Austin, 201 E 24th St, Austin, TX 78712, USA; Oden Institute for Computational Engineering and Sciences, University of Texas at Austin, 201 E 24th St, Austin, TX 78712, USA; Department of Machine Learning, Carnegie Mellon University, 5000 Forbes Avenue, Pittsburgh, PA 15213, USA; Department of Machine Learning, Carnegie Mellon University, 5000 Forbes Avenue, Pittsburgh, PA 15213, USA; Department of Computer Science, Vanderbilt University, 1400 18th Ave S, Nashville, TN 37212, USA; Research and Exploratory Development Department, Johns Hopkins University Applied Physics Laboratory, 11100 Johns Hopkins Rd, Laurel, MD 20723, USA

**Keywords:** neurosymbolic AI, scaling laws, bitter lesson, large language models, foundation models

## Abstract

The recent progress in machine learning has shifted the trends in artificial intelligence (AI) toward an overreliance on increasing amounts of data, computing power, and model parameters. These trends have resulted in success, but have also created a monolithic perspective for AI, increased the barriers to entry outside of large tech companies, and raised concerns about computational sustainability. Neurosymbolic AI is a growing area that promotes methodological heterogeneity and aims to push the frontiers of AI through affordable data and computing power.

## Introduction

State-of-the-art foundation models, such as the Gemini Ultra model from Google, contain over 1 trillion parameters. The accelerated growth of these models, along with the improvement in their performance over the past few years, has been coined by the so-called scaling laws of artificial intelligence (AI).

This growth translates into prohibitive costs for the training and use of AI. It is not sustainable, as evidenced by the excessive energy consumed and the carbon footprint resulting from these models. For example, the data centers used for the training and inference of AI account for up to 3.7% of global greenhouse emissions (1). Despite these concerns, large companies continue to rely on scaling laws as part of a profitable business model that also leads to a gatekeeping AI, whereby the training and use of state-of-the-art AI models exceeds the resources available to researchers outside of large technology companies, with estimates for training Gemini Ultra reported at $191 million (2). This gatekeeping of AI is particularly problematic, given that companies tend to release their models in closed-source form. As a result, there are growing concerns over the trustworthiness of these opaque models that are being proliferated far and wide.

Contemporary schools of thought, such as the bitter lesson popularized by Richard Sutton (3), align with the foregoing scaling laws. Sutton argues that, historically, general-purpose methods that scale with increased computation have consistently outperformed AI solutions relying heavily on human domain knowledge, going as far as to say that the only thing that matters, in the long run, is the leveraging of computation. This view has been reinforced in practice over the last few years, with the latest wave of large reasoning models, such as OpenAI's o3 model, scaling both training- and inference-time compute.

However, the question remains as to whether the current and costly scaling trends are necessary for the future of AI. At any rate, biology has taught us a different lesson: The human brain achieves data-efficient intelligence using low-power computation, operating on approximately 20 W of power (4). Assuming 24-h use over 18 yr, that is equivalent to 3.15 MWh of energy. In contrast, GPT-3 needed 1.287 GWh of training (1). In other words, the efficiency of the human brain in learning and inferencing is much greater than that of modern large models while also exceeding their cognitive capabilities and generalizing from far fewer data points.

In this brief, we argue for upending the prevailing monolithic perspective in modern AI characterized by scaling laws. Although the perspectives of scaling laws and the bitter lesson emphasize the importance of computation and data in driving long-term progress of neural networks, we believe that other innovations, including the integration domain knowledge as symbolic structures and reasoning, will significantly improve such models by making them much more efficient in terms of data, parameters, and power. We posit a practical high-level direction in the form of neurosymbolic AI, which spans the gamut of machine learning (ML) and has experienced growing interest in recent years. As we demonstrate, the constituent integration of data-driven neural methods and classical symbolic approaches has tremendous potential in reducing the scale of AI models. Moreover, the synergy of neural and symbolic methods lets us tackle tasks that purely neural methods find difficult (e.g. reliably handling compositional reasoning, real-world knowledge constraints, or interpretability demands) with a more efficient use of computation.

Of course, scale is not inherently problematic, and we have seen similar scaling laws in the logic and circuit design community be tremendously successful, with the obvious historical examples of Moore's law and Dennard scaling (5, 6). While Moore's law predicted that the number of transistors on a chip would double approximately every 2 yr, the modern AI scaling laws are observing a similar doubling of parameters at a faster rate, approximately every 6 mo (7). However, Moore's law had the complementary Dennard scaling to bound energy, with the performance per watt scaling exponentially, while the power density stayed mostly constant for decades by scaling down voltage and current as Moore's law scaled up the number of transistors. AI scaling laws have no such counterbalance. In this brief, we posit the integration of symbolic methods as that counterbalance and provide a vision for the resulting neurosymbolic AI to mitigate the deleterious effects of the current inefficient scaling laws.

This paper is organized as follows. In Classical ML assumptions and neurosymbolic AI, we begin with a brief overview of some fundamental principles on which ML relies for effective use and how scaling up data and parameters leverages these principles for improved performance. We then present arguments for how neurosymbolic AI can act as a complementary alternative to such scaling by integrating symbolic knowledge into neural models (Neurosymbolic AI: from symbols to neurons) and extracting symbolic representations from neural models (Neurosymbolic AI: from neurons to symbols). These directions of going from symbols to neurons and neurons to symbols establishes a feedback loop that allows neurosymbolic methods to efficiently handle difficult optimization landscapes for which current AI models rely on scaling laws. We present neurosymbolic methods for the development of small models in Neurosymbolic AI for small data and models and offer a psychological perspective of neurosymbolic AI in A psychological perspective of neurosymbolic AI. Given these perspectives, we revisit the topic of scaling laws and provide a nuanced view on the ways in which scaling up models and incorporating symbolic knowledge are not mutually exclusive, but rather are complementary, with an overreliance on either method being antithetical to the efficient and generalizable AI that we envision. We provide concluding remarks in Revisiting scaling laws and the bitter lesson.

## Classical ML assumptions and neurosymbolic AI

Neurosymbolic AI is the branch of AI that seeks to integrate data-driven neural networks and symbolic reasoning methods (8, 9). Symbolic methods provide semantically meaningful structures (e.g. logic-based representations, knowledge graphs, differential equations) that can be integrated into data-driven approaches. We proceed with the crux of this integration and its benefits in reducing the scale of data, parameters, and computation required by AI while preserving generalization.

The reasons for why and how neurosymbolic AI may mitigate the inefficiency of modern scaling laws trace back to the principles of ML, including the manifold hypothesis and the universal approximation theorem, upon which these laws rely (10, 11). The manifold hypothesis, for example, states that if the high-dimensional data used to train AI models shares simple features (e.g. all faces have eyes), then the learned data representation will be a combination of smooth, or flat, manifolds (i.e. non-linear surfaces) in a space of lower dimension than the data. Neural networks are effective at traversing smooth manifolds with no or few sharp regions, as they are less likely to converge to poor local optima. The universal approximation theorem states that if the neural network has enough parameters, it can be trained to find a suitable manifold representation. Therefore, the key enabling factor is the smoothness of the manifold, with current models requiring large amounts of data and parameters in order to promote smoothness for effective learning (12). However, such sharpness seems unavoidable in practice, as evidenced by the proliferation of sharpness-aware training algorithms (13). Neurosymbolic AI can address this fundamental challenge of sharpness through an alternative perspective compared with the current merely data-driven approaches.

**Fig. 1.**
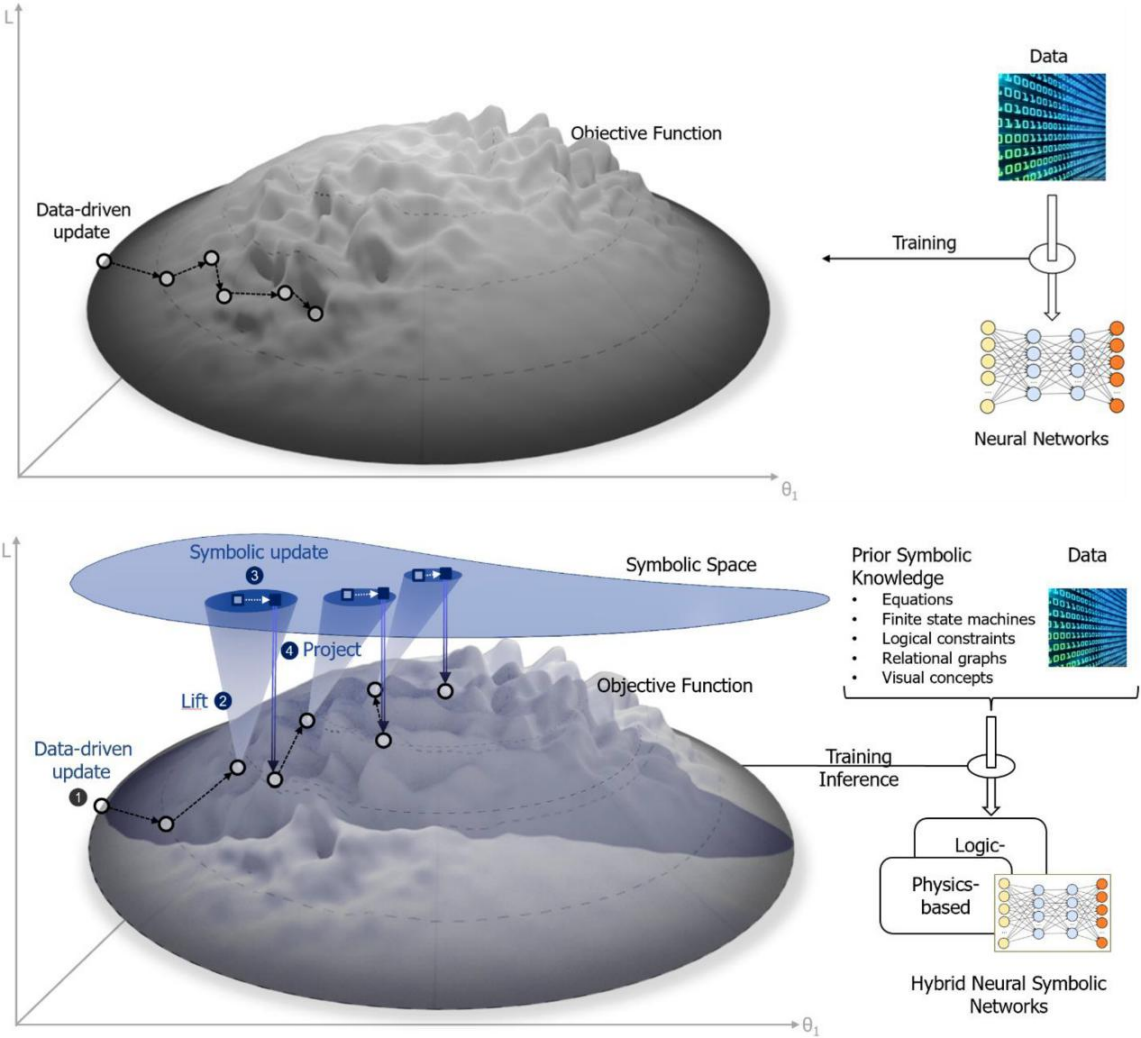
Data-driven learning on a manifold (top). Data-driven learning and symbolic reasoning on a manifold (bottom). Conventional data-driven updates are followed with symbolic reasoning by lifting the neural representation to a symbolic one. After symbolic reasoning, the symbolic representation is projected back into a neural representation. The process repeats for efficient training and inference.

Figure 1 (top) provides a rendering of the training process. The gray hill depicts an objective function (loss function) with respect to the parameters (or weights) of the neural network. Each dot on the gray hill is a point in this parametric space and represents the value of the objective function with respect to the current parametric configuration of the network. Conventional ML with neural networks entails moving in the direction of the gradient to optimize the objective function. The optimal configuration represents a best fit for the training data. The representation, while being a good fit to the underlying training data, remains agnostic about the causality or the underlying mechanisms that produced the data. In the absence of any knowledge about the underlying mechanisms, inference tasks remain bound by the distribution of the training data and struggle to generalize beyond the training data distribution.

As Figure 1 (bottom) depicts, neurosymbolic AI models would help efficiently traverse such high-dimensional manifolds through liftings of the parameter space around sharp regions into symbolic representations, enabling symbolic computation and subsequent projections back onto a smooth submanifold. This iterative interleaving of neural and symbolic representations during training based on the sharpness of the manifold naturally decomposes the learning problem into a set of smooth submanifolds separated by regions with sharpness. Small neural networks would suffice to learn on the smooth submanifold, whereas symbolic computation would be used to traverse the regions with sharpness. The overall impact would be a reduction in the amount of data, parameters, and computation required compared with current data-driven methods, which attempt to address the inevitability of sharpness by scaling up to promote smoothness.

The resulting neurosymbolic systems may not even have to abide by the manifold hypothesis that has been relied upon until now for the successes in ML, which would enable the AI community to develop much more data-efficient learning, as it is the sharp regions of the manifold that require excessive data for conventional ML to overcome or excessive parameters to smoothen (14).

While the focus of this paper has been on the conventional definition of scaling laws pertaining to the training phase of AI models, it is worth noting that scaling up inference- or test-time computation has become a very effective trend in Large Reasoning Models, in which techniques like chain-of-thought reasoning play an important role in optimizing the prompts used to interface with the trained model.

## Neurosymbolic AI: From symbols to neurons

Perhaps the most common integration of symbolic human-derived knowledge into AI models comes from the physics-informed ML community, in which physics-informed neural networks (15), physics-informed neural operators (16), and differentiable simulation (17) have all leveraged the differentiability of physics knowledge in the form of partial differential equations (PDEs) for various complex phenomena, such as predictive climate modeling (17) and predicting the dynamics of deformation (18). Due to their differentiability, these equations can be easily integrated into the loss function of the AI model, as is standard in physics-informed neural networks.

Alternatively, one can also parameterize the terms in these equations using small neural networks for each term, thereby incorporating human domain knowledge in the form of PDEs while mitigating for errors in the same through data-driven generalization. Such is the approach taken by the neural constitutive laws (18), where the preceding framework is integrated within differentiable simulations that define a visual loss function between the generated simulation that leverages the aforementioned parameterized PDEs and ground truth video data. The power of this neurosymbolic framework is evidenced by its ability to generalize to new geometries and different initial and boundary conditions not seen during training. By introducing these symbolic physical priors as an inductive bias, the neural network can satisfy conservation laws with minimal training data consisting of a single video capturing the relevant dynamics, a feat that was not possible with the competing purely neural approaches. Physics knowledge has also been integrated into popular AI-based rendering methods, such as neural radiance fields, which have been augmented with physics-based material point methods to enforce that the conservation laws of mechanics are followed by the rendered result while also accelerating the rendering process by 2 orders of magnitude compared with neural radiance field baselines (19).

Another increasingly popular trend for incorporating symbolic representations into neural models is to leverage knowledge graphs, which act as graph representations of semantic relations across objects, to improve accuracy and reduce the amount of training data (20). For example, Amazon uses a large-scale knowledge graph in synergy with their large language models (LLMs) (21). Their COSMO framework leverages LLMs to construct common-sense knowledge graphs from customer interaction data, enabling Amazon's recommendation engine to infer relationships between products and their human contexts, such as functions or audiences. This knowledge is typically derived manually, but recent methods, such as GraphRAG (22), automatically extract knowledge graphs from a corpora of text data to augment the text generation capabilities of LLMs by incorporating the extracted knowledge graph into the retrieval-augmented generation process. Unlike conventional deep learning approaches that combine data, representation, and downstream tasks into one set of parameters that are difficult to disentangle, knowledge graphs allow models to separate these components. Consequently, when creating a knowledge graph for one problem, it can be reused later on for other tasks, as it is independent of the problem. Furthermore, they are interpretable and can be easily expanded by users as needed, thereby growing over time and extending the capabilities of all models related to it.

Recent work, such as Zhang et al. (23), highlights the limitations of pure scaling, as they reveal that large neural models lack true reasoning abilities and instead can tend to rely on statistical data patterns that do not suffice for generalizing within the same family of reasoning tasks, much less out-of-distribution problems. Consequently, several methods have explored the update of neural network parameters using projectors from symbolic representations. For example, in Ahmed et al. (24), logical constraints are translated into differentiable loss functions that are used during neural model training that enables the embedding of symbolic knowledge within neural networks while maintaining full differentiability.

## Neurosymbolic AI: From neurons to symbols

Extracting symbolic representations from neural networks that span a continuous topological space is an open area of research, often referred to as symbolic distillation (25). Some popular examples include the use of quantized ML models, such as vector quantized variational auto-encoders (26, 27), vector quantized diffusion models (28), QLoRA (Quantized Low-Rank Adaptation) (29), and quantified bottleneck insertion (30, 31), to learn neural network representations whose weights take on categorical values, such as integers or binary values, as opposed to the conventional continuous-valued representations learned by conventional neural networks. Intuitively, such quantized representations enable neural networks to learn symbolic representations. Indeed, consider a learned representation whose *n* weights or activations are binary. One can consider the $2^{n}$ weight or activation values as the possible atomic propositions in some logic or the letters in the vocabulary of a symbolic language. Then, the observations of these atomic propositions can be used to extract symbolic graph representations, such as deterministic finite automata and knowledge graphs, by using classical techniques like grammatical inference. Such methods have been used successfully in (31) to learn a quantized representation of recurrent neural networks and then using those quantized weights or activations to learn a finite state machine that is much smaller than the corresponding recurrent neural network and captures relevant dynamics for the same. Such quantization is also useful for improving the efficiency of AI models by enabling the weights of neural networks to be represented using much fewer bits, as evidenced by QLoRA in quantizing weights down to 4 bits (29).

Other methods have explored automated fine-tuning frameworks using feedback signals from formal verification, uncertainty quantification, and disentanglement of the outputs of foundation models (32). These methods uplift neural representations into a symbolic space and obtain a measure of compliance with task descriptions. This measure is then directly used to optimize neural model parameters. For example, the end user can provide formal specifications that a given generative AI model should satisfy. By using model checking techniques for formal verification, it is possible to verify how many of the properties are satisfied by the system and rank order responses from the AI model based on that. That rank ordering can then be used as a type of reward signal to fine-tune the model so that it generates outputs more likely to satisfy the given properties. One can also attempt to learn the symbolic representations from LLMs by treating the model as an oracle and leveraging grammatical inference techniques (33).

## Neurosymbolic AI for small data and models

In the realm of language modeling and reasoning, there is ample evidence that neurosymbolic AI approaches can significantly improve data and parameter efficiency. For example, symbolic knowledge distillation has been used to distill common sense knowledge from GPT-3 into a common sense knowledge graph and a corresponding neural model that are 100 smaller than GPT-3 while achieving superior common sense reasoning performance (34). Similarly, the neurosymbolic framework Ctrl-G (35) takes symbolic logic constraints as inputs and integrates them into the distillation of a small LLM such that its generated text satisfies the aforementioned constraints. Ctrl-G can be used to distill any production-ready LLM into a tractable probabilistic model (e.g. a hidden Markov model) that supports efficient reasoning about logical constraints encoded as deterministic finite automata. Indeed, in a human evaluation study on the quality of generated text and its abidance to the given logical constraints, the Ctrl-G model consisting of a 7B-parameter LLM for text generation and the distilled 2B-parameter hidden Markov model for symbolic reasoning outperformed by over 30% the much larger 175B-parameter GPT-3.5 and GPT-4 models, the latter of which is rumored to be over 1 trillion parameters. In contrast, models based on neural methods for distillation tend to underperform their larger counterparts (36).

While the preceding methods for neurosymbolic distillation from large models into small ones are promising for the development of efficient models, these methods presuppose that the large models have already been trained, which is useful for leveraging the sizeable investments industry has made into developing large models. However, we envision that neurosymbolic AI will make it so that we do not have to train these large models to begin with. More specifically, symbolic knowledge should effectively and robustly fill the gaps at training and inference time, so that much fewer data and parameters would be needed. To that end, recent methods have explored the novel integration of physics and conservation laws within neural models (37). Rather than distilling physics laws into a neural network, this system involves co-designing neural networks for estimating physical variables and the symbolic equations that govern conservation laws in off-road autonomous driving. The system is able to match the performance of the state of the art in predicting the motion of the vehicle with just 0.1% of the training time and 1% of the training data, while requiring 96.9% fewer parameters. With further training, this approach achieves a 46% performance improvement over the state of the art.

Neurosymbolic AI also efficiently enables human subject matter experts to provide a small number of examples, which can be seamlessly integrated into the symbolic components of the model (e.g. via knowledge graphs) and are then used to more efficiently train the neural network component (38). The trained network can then utilize the symbolic knowledge as a crucial part of its reasoning process, combining the best of both worlds: fast adaptation to novel situations from symbolic reasoning and generalization capabilities in high-dimensional spaces from ML. Through this integration of symbolic knowledge, neurosymbolic methods result in smaller models. In one such setup, utilizing symbolic knowledge for few-shot learning of novel objects results in models that are approximately 95% smaller compared with the best neural-only baseline while still outperforming it by 2% (38).

## A psychological perspective of neurosymbolic AI

Human cognitive systems face a similar challenge as AI systems. Expertise requires a substantial amount of time and experience, but it is time-consuming and impractical to do this with trial and error. For example, learning to drive a car involves mastering a substantial number of rules for traffic and car operation as well as driving-specific perceptual and motor skills. Furthermore, a driver may need to drive in new contexts (e.g. a country with different traffic laws) and would not have time to relearn their driving skills from scratch. In AI terms, the learning challenge involves a high-dimensional space, sparse training data, intermittent and possibly delayed rewards, unexpected distribution shifts, and the need for few-shot learning. How does human cognition solve this challenge?

One common theme that has emerged from the past 50+ years of cognitive psychology is that of dual process theories, which posit that there are 2 modes of knowledge representation and execution, variously termed subsymbolic/symbolic, implicit/explicit, parallel/sequential, or system 1/system 2 (39). In the first mode (subsymbolic, implicit, parallel), learning is slow and guided by trial and error. However, once the habit is acquired, it is fast and automatic and can rapidly integrate perceptual and motor information (e.g. a soccer player dribbling a ball without deliberate thought). In the second mode (symbolic, explicit, sequential), learning can be quick, but applying it can be quite slow and laborious.

Several researchers have noted that these two modes complement each other (40). Consider driving a car as a domain [for analysis of other domains, see Rousse and Dreyfus (41)]. The novice driver initially relies on symbolic knowledge and rules as training wheels or guardrails (e.g. “stay 2 seconds behind the car in front; stop when the light is red”). Application of this symbolic knowledge allows the novice driver to stay alive and continue to gain experience. The experience, in turn, slowly trains the perceptuomotor systems of the driver so that, eventually, stopping at a red light becomes automatic, and with even more experience, driving in rush-hour traffic becomes automatic as well (41). If the driver is in a context in which existing experience and symbolic knowledge does not apply (e.g. operating a car with one-pedal driving), they can induce symbolic rules and reason over them, and use that to bootstrap their learning experience [see bottom-up learning in Sun (40)].

Crucially, because symbolic knowledge can be compositional, a small number of rules can cover a vast variety of potential scenarios, similar to how a small number of words and grammatical compositional rules can apply to a vast number of sentences (42). In ML parlance, symbolic knowledge provides a compositional inductive bias for the learning agent whenever the agent is outside a familiar or known operational regime. The bias is not necessarily optimal, but it usually gets the agent into the neighborhood of optimal solutions similar to the symbolic updates in Figure 1. A related advantage is that if the agent is stuck in a local minimum, symbolic knowledge can provide guided exploration for a better solution. For example, if a novice driver has difficulty with parallel parking, they can “talk themselves” through it (similar to chain-of-thought reasoning with LLMs) using their symbolic knowledge of causal and geometric relationships.

While there has been debate about how exactly symbolic processing is realized in the human brain (43), the overall lesson for AI systems is clear: the flexibility, fluidity, and adaptability of human cognition is supported by the complementary interplay of symbolic and neural, or subsymbolic, processing.

## Revisiting scaling laws and the bitter lesson

Current foundation models rely on neural scaling laws under the assumption that given sufficient parameters, data, and compute, models can discover general and flexible representations that can outperform handcrafted knowledge. The bitter lesson is a reflection on how such assumptions have been true in retrospect, with memorable examples including the use of general purpose learning and search techniques in the AlphaGo Zero model that outperformed methods leveraging human tactics (44), as well as the shift from human-engineered features in computer vision to data-driven feature extraction (45). For the former, the system was able to discover the infamous move 37, which may not have been discovered if the bias imposed by human-derived tactics was in effect. However, a notable counterexample to such thinking includes AlphaStar (46), which incorporated human strategies into the learning process by bootstrapping the learning process from 987,000 replays from the top 22% of players. More to the point, the ablated system without the human strategies significantly underperformed both AlphaStar as well as human amateurs on StarCraft.

While the AlphaStar counterexample does not invalidate the bitter lesson (it is possible that, given sufficient training time, AlphaStar may have found novel optimal strategies), there are reasons for concern about the feasibility of continued scaling. In particular, there may not be enough data even in principle to sustain data-driven improvements in frontier models (47). There are also many domains in which data and compute are inherently limited and not readily scalable, such as AI on the edge and low size, weight, and power systems. In such scale-limited situations, it is necessary to complement the neural model with strong inductive biases.

Furthermore, while the bitter lesson argues for AI agents that can discover like we can, not which contain what we have discovered, the integration of human-derived rules (inductive biases) and the discovery and refinement of new knowledge are not mutually exclusive. By analogy, if a child is unable to solve a multidigit addition problem, a symbolic hint like “carry the 1” can guide them into a learning space in which they can productively generalize (e.g. “carry the 2”). Indeed, we noted examples of neurosymbolic systems that demonstrate both discovery and efficient use of prior knowledge, such as the neural constitutive laws (18) whereby human-discovered PDEs are parameterized to be used as inductive biases (such biases can also be used train networks that outperform models one hundred times their size while using 1% of the data) (37). The field of physics-informed ML, and of human learning more generally, showcases how such approaches are complementary rather than contradictory.

Finally, there is the more fundamental problem of whether logical reasoning, which is broadly considered a precondition for artificial general intelligence, can be learned from data given sufficient computation. In particular, current AI models can attain near-perfect accuracy in one distribution of reasoning problems but are unable to generalize to other distributions in the same problem space (23). The evidence indicates that such models do not learn to reason, but rather simply learn statistical patterns that best fit the training data (48, 49). While such models have proven tremendously useful for applications such as code generation and text summarization, the evidence to date suggests that scaling, by itself, may not suffice to endow models with sophisticated and rigorous reasoning expertise.

In summary, we advocate that discovery and knowledge incorporation are not mutually exclusive. It is crucial to have AI agents that can discover like we can, but part of what it means to discover like people is leveraging and refining pre-existing knowledge to facilitate discovery. As we have alluded to, the key issue is the nature of the inductive bias: how it is expressed, how it guides the learning and inference of the AI model, and how it can complement data-driven discovery. At a high level, we have suggested in this brief a hybrid architecture in which symbolic reasoning can be interleaved with neural computation to reduce scale as follows:

- Data-driven representations provide powerful baseline capabilities to the neural system.
- Translation, or lifting, of neural representations to symbolic forms for logical reasoning.
- Projection of symbolic representations back to neural space for continued learning.
- Symbols and associated relationships can be discovered by the neural system from data-driven representations (e.g. visual features), incorporated through domain-specific constraints (e.g. semantics of traffic signs), or refined through exploration guided by symbolic reasoning (e.g. identifying parking violations based on street signs).
- The symbolic system could also incorporate distilled knowledge from larger neural models, enabling smaller, more efficient models.

The previous approach creates a feedback loop, in which symbolic reasoning guides the neural learning process toward more efficient solutions and leverages the benefits of data and compute scaling while addressing its limitations. Therefore, we see our vision for neurosymbolic AI as complementary to the scaling laws of modern AI while simultaneously providing an avenue to upend the gatekeeping of state-of-the-art AI that has resulted from the inefficient adoption of these same laws.

## Conclusion

The scaling laws of recent years have produced impressive results in vision and language. However, this process of developing increasingly costly models is unsustainable and introduces a gatekeeping of AI by those entities that can afford the development of such models. Neurosymbolic AI is emerging as a conduit to upend the deleterious effects of these scaling laws. While some agencies have recognized the potential of neurosymbolic AI with investments in this space (50), a more concerted and interdisciplinary effort is necessary to balance the scaling laws and enable the advent of efficient and trustworthy AI systems.

## Data Availability

All data are included in the manuscript and/or supporting information.

## References

[1] Cho R . AI’s growing carbon footprint. Columbia Climate School. State of the Planet, June 9, 2023 [accessed 2024 Dec 15]. https://news.climate.columbia.edu/2023/06/09/ais-growing-carbon-footprint/.

[2] Maslej N, et al The AI index 2024 annual report. AI Index Steering Committee. Institute for Human–Centered AI, Stanford University, Stanford, CA, April 2024 [accessed 2024 Dec 15]. https://hai.stanford.edu/ai-index/2024-ai-index-report.

[3] Sutton R . 2019. The Bitter Lesson. 2019 [accessed 2024 Dec 15]. http://incompleteideas.net/IncIdeas/BitterLesson.html.

[4] Hofman MA . 2014. Evolution of the human brain: when bigger is better. Front Neuroanat. 8:15. 24723857 10.3389/fnana.2014.00015 PMC3973910

[5] Moore GE . 1998. Cramming more components onto integrated circuits. Proc IEEE Inst Electr Electron Eng. 86(1):82–85.

[6] Dennard RH, et al 1974. Design of ion-implanted MOSFET’s with very small physical dimensions. IEEE J Solid-State Circuits. 9(5):256–268.

[7] Pilz K, Heim L, Brown N. 2025. Increased compute efficiency and the diffusion of AI capabilities. In: *Proceedings of the AAAI Conference on Artificial Intelligence*. Vol. 39.

[8] d’Avila Garcez A, Lamb LC. 2023. Neurosymbolic AI: the 3rd wave. Artif Intell Rev. 56(11):12387–12406.

[9] Besold TR, et al 2021. Neural-symbolic learning and reasoning: a survey and interpretation 1. In: Hitzler P, Sarker MK, editors. Neuro-symbolic artificial intelligence: the state of the art. Amsterdam, the Netherlands: IOS Press. p. 1–51.

[10] Fefferman C, Mitter S, Narayanan H. 2016. Testing the manifold hypothesis. J Amer Math Soc. 29(4):983–1049.

[11] Hornik K, Stinchcombe M, White H. 1989. Multilayer feedforward networks are universal approximators. Neural Netw. 2(5):359–366.

[12] Gamba M, Englesson E, Björkman M, Azizpour H. 2023. Deep double descent via smooth interpolation. *Transact Mach Learn Res.* https://openreview.net/forum?id=fempQstMbV.

[13] Foret P, Kleiner A, Mobahi H, Neyshabur B. 2020. Sharpness-aware minimization for efficiently improving generalization. In: *International Conference on Learning Representations*.

[14] Kiani BT, Wang J, Weber M. 2024. Hardness of learning neural networks under the manifold hypothesis. *Adv Neural Inf Process Syst.* 37:5661–5696.

[15] Raissi M, Perdikaris P, Karniadakis GE. 2019. Physics-informed neural networks: a deep learning framework for solving forward and inverse problems involving nonlinear partial differential equations. J Comput Phys. 378:686–707.

[16] Li Z, et al 2024. Physics-informed neural operator for learning partial differential equations. ACM/IMS J Data Sci. 1(3):1–27.

[17] Liang J, Lin MC. 2020. Differentiable physics simulation. In: *ICLR 2020 workshop on integration of deep neural models and differential equations*.

[18] Ma P, et al 2023. Learning neural constitutive laws from motion observations for generalizable PDE dynamics. In: *International Conference on Machine Learning*, PMLR, p. 23279–23300.

[19] Li X, et al PAC-NeRF: Physics augmented continuum neural radiance fields for geometry-agnostic system identification. In: *The eleventh international conference on learning representations*.

[20] An B, Chen B, Han X, Sun L. 2018. Accurate text-enhanced knowledge graph representation learning. In: *Proceedings of the 2018 Conference of the North American Chapter of the Association for Computational Linguistics: Human Language Technologies, volume 1 (long papers)*, p. 745–755.

[21] Yu C, et al COSMO: a large-scale e-commerce common sense knowledge generation and serving system at Amazon. 2024 [accessed 2024 Dec 15]. https://www.amazon.science/publications/cosmo-a-large-scale-e-commerce-common-sense-knowledge-generation-and-serving-system-at-amazon

[22] Edge D, et al 2024. From local to global: a graph rag approach to query-focused summarization. arXiv 2404.16130. 10.48550/arXiv.2404.16130, preprint: not peer reviewed.

[23] Zhang H, Li LH, Meng T, Chang K-W, Van Den Broeck G. 2023. On the paradox of learning to reason from data. In: *Proceedings of the Thirty-second International Joint Conference on Artificial Intelligence*, p. 3365–3373.

[24] Ahmed K, Teso S, Chang K-W, Van den Broeck G, Vergari A. 2022. Semantic probabilistic layers for neuro-symbolic learning. Adv Neural Inf Process Syst. 35:29944–29959.

[25] Acharya K, Velasquez A, Song HH. 2024. A survey on symbolic knowledge distillation of large language models. IEEE Trans Artif Intell. 5(12):5928–5948.

[26] Van Den Oord A, Vinyals O, Kavukcuoglu K. 2017. Neural discrete representation learning. *Adv Neural Inf Process Syst.* 30.

[27] Razavi A, Van den Oord A, Vinyals O. 2019. Generating diverse high-fidelity images with vq-vae-2. *Adv Neural Inf Process Syst.* 32.

[28] Gu S, et al 2022. Vector quantized diffusion model for text-to-image synthesis. In: *Proceedings of the IEEE/CVF Conference on Computer Vision and Pattern Recognition*, p. 10696–10706.

[29] Dettmers T, Pagnoni A, Holtzman A, Zettlemoyer L. 2023. Qlora: efficient finetuning of quantized llms. *Adv Neural Inf Process Syst.* 36:10088-10115.

[30] Koul A, Fern A, Greydanus S. 2019. Learning finite state representations of recurrent policy networks. In: *International conference on learning representations*.

[31] Carr S, Jansen N, Topcu U. 2021. Task-aware verifiable RNN-based policies for partially observable Markov decision processes. J Artif Intell Res. 72:819–847.

[32] Yang Y, et al 2024. Fine-tuning language models using formal methods feedback: a use case in autonomous systems. Proc Mach Learn Syst. 6:339–350.

[33] Chen L, Trivedi A, Velasquez A. 2024. LLMs as probabilistic minimally adequate teachers for DFA learning. arXiv 2408.02999. 10.48550/arXiv.2408.02999, preprint: not peer reviewed.

[34] West P, et al 2022. Symbolic knowledge distillation: from general language models to commonsense models. In: *Proceedings of the 2022 Conference of the North American Chapter of the Association for Computational Linguistics: Human Language Technologies*, p. 4602–4625.

[35] Zhang H, Kung P-N, Yoshida M, den Broeck GV, Peng N. 2024. Adaptable logical control for large language models. *Adv Neural Inf Process Syst.* 37: 115563–115587.

[36] Xu X, et al 2024. A survey on knowledge distillation of large language models. arXiv 2402.13116. 10.48550/arXiv.2402.13116, preprint: not peer reviewed.

[37] Zhao Z, Li B, Du Y, Fu T, Wang C. 2024. PhysORD: a neuro-symbolic approach for physics-infused motion prediction in off-road driving. In: *2024 IEEE/RSJ International Conference on Intelligent Robots and Systems (IROS)*.

[38] Bhagat S, Stepputtis S, Campbell J, Sycara K. 2023. Sample-efficient learning of novel visual concepts. In: *Conference on Lifelong Learning Agents*, PMLR, p. 637–657.

[39] Sun R . 2024. Dual-process theories, cognitive architectures, and hybrid neural-symbolic models. NeSy AI. 1:1–9. 10.3233/NAI-240720

[40] Sun R . 2013. Autonomous generation of symbolic representations through subsymbolic activities. Philos Psychol. 26(6):888–912. 10.1080/09515089.2012.711035

[41] Rousse BS, Dreyfus S. 2021. Revisiting the six stages of skill acquisition. In: Silva Mangiante E, Peno K, Northup J, editors. Teaching and learning for adult skill acquisition: Applying the Dreyfus & Dreyfus model in different fields. Charlotte (NC): Information Age. p. 3–30.

[42] Dekker RB, Otto F, Summerfield C. 2022. Curriculum learning for human compositional generalization, Proc Natl Acad Sci U S A. 119(41):e2205582119. 10.1073/pnas.2205582119 36191191 PMC9564093

[43] Marcus GF . 2001. The algebraic mind: integrating connectionism and cognitive science. Learning, development, and conceptual change. Cambridge (MA): MIT.

[44] Silver D, et al 2017. Mastering the game of go without human knowledge. Nature. 550(7676):354–359. 29052630 10.1038/nature24270

[45] Nixon M, Aguado A. 2019. Feature extraction and image processing for computer vision. Cambridge (MA): Academic Press.

[46] Vinyals O, et al 2019. Grandmaster level in StarCraft II using multi-agent reinforcement learning. Nature. 575(7782):350–354. 31666705 10.1038/s41586-019-1724-z

[47] Villalobos P, et al 2024. Position: will we run out of data? Limits of LLM scaling based on human-generated data. In: *ICML'24: Proceedings of the 41st International Conference on Machine Learning*. p. 49523–49544.

[48] Valmeekam K, Stechly K, Gundawar A, Kambhampati S. 2024. Planning in strawberry fields: evaluating and improving the planning and scheduling capabilities of LRM o1. arXiv 2410.02162. 10.48550/arXiv.2410.02162, preprint: not peer reviewed.

[49] Wang K, et al On the planning abilities of OpenAI’s o1 models: feasibility, optimality, and generalizability. In: *Language Gamification-NeurIPS 2024 Workshop*.

[50] Velasquez A, Neema S. Assured neuro symbolic learning and reasoning (ANSR). *Defense Advanced Research Projects Agency (DARPA*).

